# Green electrosynthesis of drug metabolites

**DOI:** 10.1093/toxres/tfad009

**Published:** 2023-03-07

**Authors:** Ridho Asra, Alan M Jones

**Affiliations:** Molecular Synthesis Laboratory, School of Pharmacy, University of Birmingham, Edgbaston, Birmingham B15 2TT, United Kingdom; Molecular Synthesis Laboratory, School of Pharmacy, University of Birmingham, Edgbaston, Birmingham B15 2TT, United Kingdom

**Keywords:** metabolite, electrosynthesis, drug

## Abstract

In this *concise* review, the field of electrosynthesis (ES) as a green methodology for understanding drug metabolites linked to toxicology is exemplified. ES describes the synthesis of chemical compounds in an electrochemical cell. Compared to a conventional chemical reaction, ES operates under green conditions (the electron is the reagent) and has several industrial applications, including the synthesis of drug metabolites for toxicology testing. Understanding which circulating drug metabolites are formed in the body is a crucial stage in the development of new medicines and gives insight into any potential toxic pathologies resulting from the metabolites formed. Current methods to prepare drug metabolites directly from the drug molecule often involve time-consuming multistep syntheses. Throughout this review, the application of green ES to (i) identify drug metabolites, (ii) enable their efficient synthesis, and (iii) investigate the toxicity of the metabolites generated are highlighted.

## Introduction

High-throughput screening of small molecules is a key step in identifying potent compounds that can be further refined through the hit-to-lead stage of drug design. In selecting a compound with the most promise for further development, toxicity screening prior to and during lead optimization is essential to avoid unwanted traits in the final drug candidate.

Despite a variety of different strategies being developed to predict the toxicity profile of small molecules in preclinical studies, many drugs have failed during clinical studies and only a limited number of new drugs are approved for market authorization each year.^1^ Ominously, a >90% failure rate of new chemical entities (NCE) can be expected during the drug development process.^2^ The Food and Drug Administration (FDA) has approved an average of 43 new drugs annually between 2012 and 2021.^3^ Despite a drug receiving regulatory approval, unexpected adverse reactions that were not observed during clinical trials can also lead to market withdrawal of the drug.^4^ Retrospective studies on cases of unexpected adverse drug reactions are well known.^5^^,^^6^ Thus, toxicology screening is paramount at all stages of drug development.

Mainstream toxicological screening tools do not accurately mimic all aspects of metabolism in humans.^7^^,^^8^ Moreover, a transient human metabolite^9^ can be challenging to identify in traditional toxicology studies. To understand drug metabolites and their safety, both the chemical structure and systemic exposure are investigated to evaluate the toxicological significance. Metabolites accounting for >10% of total drug-related exposure at steady state must be assessed in safety studies, particularly for drug metabolites present at a disproportionate level.^10^ Studies to assess the toxicity of potential human metabolites are costly, time consuming, and are also limited by the availability of sufficient samples for testing.

Electrosynthesis (ES) is the synthesis of chemical compounds in an electrochemical cell, consisting in the simplest sense of a galvanic cell, an electrochemical analyzer, and 2 main electrodes in a conducting solution. Compared to purely chemical redox reactions, ES can be a greener approach without the need for additional chemical reagents (as the electron is the reagent), offering improved or different selectivity to traditional approaches.

ES is a cutting-edge development in the field of toxicology screening. ES, as a methodology, can prepare new functionality onto existing drug molecules, providing an alternative drug metabolite synthesis. This approach mimics the natural phase I metabolism process of a drug molecule in the body. Using mild oxidation conditions, in contrast to traditional chemical synthesis, ES can play a role in generating the metabolic products of a new chemical entity. ES is a powerful platform to activate and functionalize small organic molecules, performing a redox reaction by adding or removing electrons under controlled voltage or controlled current conditions through a conductive solution to convert a substrate directly on the electrode surface or mediated in-solution approaches.^11^ ES offers a mild, safe, green, and promising alternative to conventional synthetic processes without the need of chemical REDOX reagents or the use of protective groups in concession steps.^12–14^ ES is designated as a *green chemistry* platform** because electrons are a renewable resource. ES satisfies 9 of the 12 postulates of green chemistry, such as green solvents, less hazardous chemical synthesis process, designing safer chemicals, preventing waste production, improved atom economy, energy efficiency, real-time analysis, synthetic catalytic processes, and reducing the use of derivatives/protecting groups.^14–16^

**Fig. 1:**
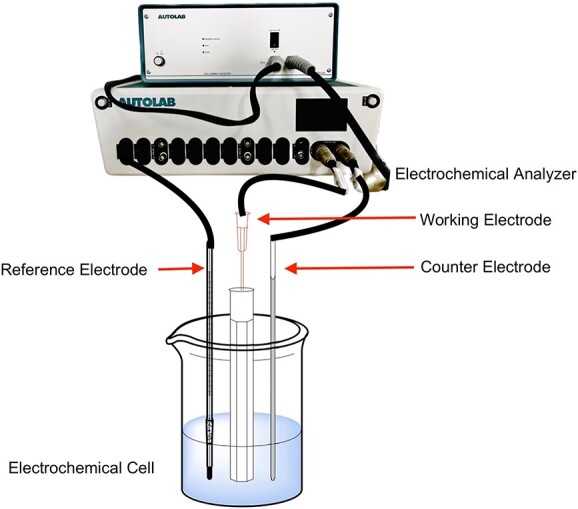
Key components of an ES setup: electrochemical analyzer,  reference electrode, working electrode, and counter electrode.^17^

An ES setup consists of an electrochemical cell in tandem with an electrochemical analyzer such as a potentiostat, galvanostat, or impedance analyzer. Using a 3-electrode setup comprising the working, counter, and reference electrodes (WE, CE, RE, respectively), this electrochemical device could be employed to run a cyclic voltammetry experiment to determine REDOX behavior, enable electrosynthetic preparation of drug metabolites, and gain complementary mechanistic insight into drug metabolism pathways^11^ (Fig. 1).

**Fig. 2:**
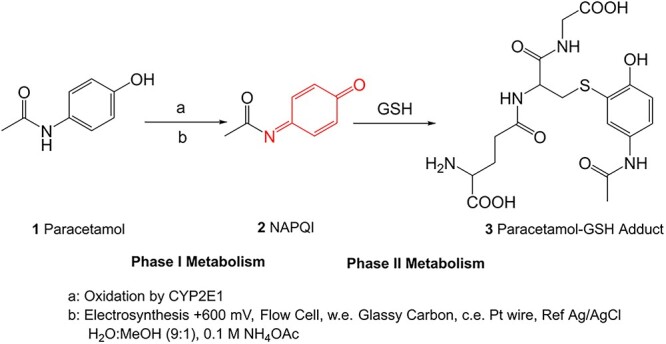
Electrosynthetic phase I and II metabolic mimicry of paracetamol oxidation and GSH adduct formation.

The ES setup can be adapted to resemble how drug metabolism occurs in the human body (Fig. 2). Paracetamol (**1**), an analgesic and antipyretic, predominantly undergoes glucuronidation and sulfation to produce stable excreted metabolites. At levels far exceeding recommended therapeutic doses, these pathways became saturated, and cytochrome P450 (CYP) enzymes oxidatively metabolize 1 to afford an electrophilic quinone imine derivative (*N*-acetyl-*para*-benzoquinone imine; NAPQI, **2**). If the body’s glutathione (GSH) levels become depleted, it is possible that **2** can accumulate. NAPQI (**2**) may then interact with cellular macromolecules, resulting in hepatoxicity.^8^^,^^18^^,^^19^ Electro-metabolism studies of **1** have successfully demonstrated that ES mimics these phase I and II metabolism reactions. ES replicates the reactive nature of the phase I metabolite by forming NAPQI (**2**) and enables trapping via conjugation with GSH (**3**).^20–24^ ES can offer an alternative method for preparing drug metabolites and gives an insight into the future applications of ES as a complementary technology in toxicology studies.

The ability to synthesize potential drug metabolites from the parent drug by ES has enabled new avenues of investigation. The capability to generate diverse drug metabolites, scalability, reproducibility, and purification of electrosynthetic drug metabolites is a key advantage of ES for toxicity studies.^11^ ES can accelerate a drug discovery rate limiting step and be a potential screening tool early in preclinical studies.

## Role of bioactivation and resulting toxicity as a key stage in the development of prospective drug candidates

Drug metabolism in the liver includes biotransformation mechanisms to inactivate the drug and enhances the resulting drug metabolite’s excretion by increasing the polarity of the compound. Such bioactivation pathways are typically divided into 2 phases, e.g. in *phase I metabolism* catalyzed by CYP-450 enzyme isoforms, drugs are subjected to chemical transformation by introducing a polar group, including (i) oxidation; (ii) reduction; or (iii) hydrolysis.^25^ This phase I metabolism yields polar metabolites. In *phase II metabolism*, the metabolite undergoes conjugation with an endogenous moiety, including (i) glucuronidation with glucuronic acid; (ii) glutathione; (iii) acetylation by acetyl-CoA; (iv) methylation of *S*-adenosylmethionine; (v) conjugation with glycine or water; or (vi) sulfation by phosphoadenosyl phosphosulfate.^26^^,^^27^ These phase II metabolic events afford products with increased water solubility. Therefore, they can be eliminated through bile or urine.^28^^,^^29^

In addition to the formation of stable metabolites in phase I, metabolic transformation has the potential to produce unstable, toxic, and reactive intermediates. Endogenous detoxifying substrates, found in phase II metabolism, can stabilize the toxic intermediate at a low concentration. These detoxifying mechanisms could be overwhelmed at higher concentrations, and the resulting toxic products may prevail. Consequently, reactive metabolites can establish covalent interactions with cellular macromolecules, e.g. proteins leading to immune response; and DNA leading to carcinogenesis; or noncovalent interactions with target molecules, e.g. lipid peroxidation generation of cytotoxic oxygen radicals, impairment of mitochondrial respiration, depletion of GSH leading to oxidative stress, modification of sulfhydryl groups impair Ca^2+^ homeostasis, and protein synthesis inhibition among others.^30^

Bioactivation pathways leading to toxophores must be determined to minimize potential safety liabilities. By implementing a structure–activity relationship (SAR) approach, lead compounds can be optimized for their intended target by modifying potential toxophore regions of the structure. Thus, pharmacokinetic and pharmacodynamics properties, as well as the safety profile, can be maintained or, indeed, improved. The bioactivation of small molecules is known to generate several reactive and toxic structural entities (Table 1), grouped into 3 major types, e.g. electron-deficient double bonds (quinones, quinone methides, quinone imines, imine methides, diimines, Michael acceptors, and electronically stabilized – iminium ions), epoxides derived from CYP-mediated oxidation of aryl rings and double-bond containing compounds, and acyl glucuronides.^31^

**Table 1:** Representative examples of chemically reactive metabolites.

#	Parent compound containing structural alerts (SAs)/ (Toxophores)	Toxic entities	Toxic metabolites	GSH depletion	Metabolic enzyme	Pathology	Ref (s).
Electron-deficient double/triple bonds							
1.	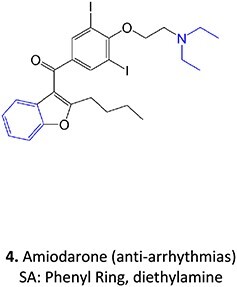	Quinone	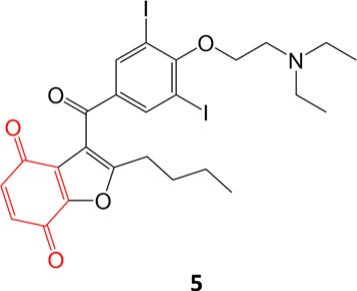	Required	CYP3A4 and CYP2C8	Immune-related toxicity	^40^
	*Other drugs that form a quinone:* raloxifene,^32^ paroxetine,^33^ methyl dopa,^34^ nefazodone,^35^ carvedilol,^36^ tadalafil,^37^ bisphenol A,^38^ doxorubicin^39^	*N-*dealkylation (aldehyde by-product)	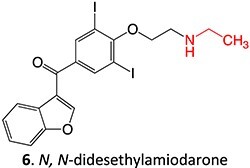	Required	CYP1A2, CYP2C9, or CYP3A4	Inhibition of CYP2D6 metabolism	^41^
2.	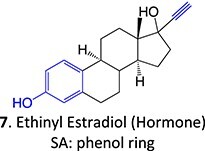	a. *o*-Quinone b. Hypothesized oxirene species	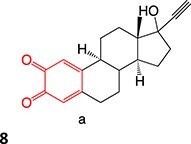 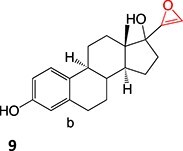	Required	CYP3A4	Alkylates the heme group or protein resulting in the mechanism-based inactivation of the isozyme	^42^ ^,^ ^43^
3.	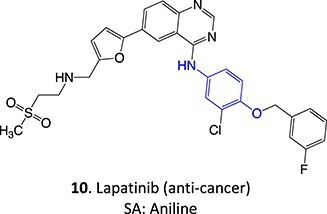	quinone imine	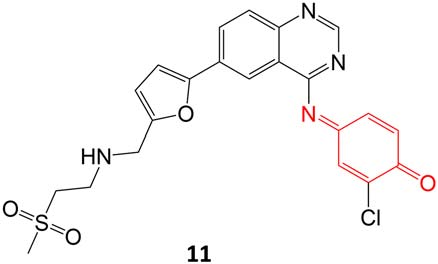	Required	CYP3A4	Hepatotoxicity	^44^
	*Other drugs that form a quinone imine reactive metabolite*: diclofenac,^45^ lumiracoxib,^46^ amodiaquine,^47^ nomifensine,^48^ (dasatinib, gefitinib, erlotinib),^49^^,^^50^ carbamazepine,^51^ atorvastatin,^52^ aripiprazole,^53^ trazodone,^54^^,^^55^ (entacapone, tolcapone),^56^^,^^57^ minocycline,^58^ vesnarinone,^59^ indomethacin,^60^ chlorpromazine^61^						
4.	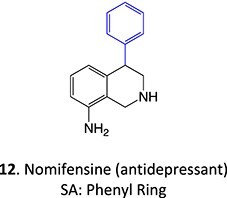	quinone methide	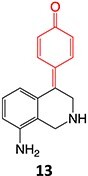	NA	CYPs	Hemolytic anemia, hepatitis	^62^
	*Other drugs that form a quinone methide:* tamoxifen,^63^^,^^64^ tacrine,^65^ troglitazone,^66^ nevirapine,^67^ levamisole,^68^ phencyclidine,^69^ eugenol,^70^ arzoxifene,^71^ ferrocifens^72^						
5.	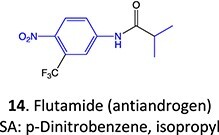	a. 2-Hydroxyflutamide b. Nitroso c. Hydroxylamine d. Bis-imine quinone	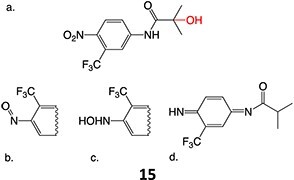	Required	CYP1A2	Inhibition of taurocholate efflux in human hepatocytes	^73^ ^,^ ^74^
6.	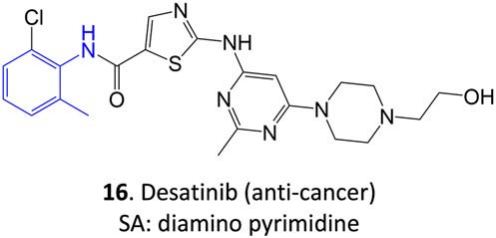	Imine methide	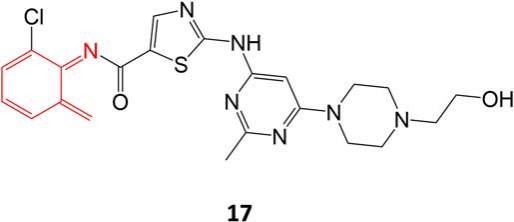	Required	CYP3A4	Hepatotoxicity	^75^
	*Other drugs that form imine methide:* trimethoprim,^76^ eltrombopag^77^						
7.	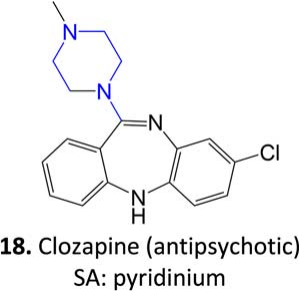	Nitrenium ion/iminium	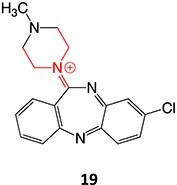	Required	CYP3A4/CYP1A2	Neutrophils apoptosis	^78^ ^,^ ^79^
	*Other drugs that form a nitrenium ion*: nomifensine,^62^ mianserin,^80^ aminopyrine^68^^,^^81^						
8.	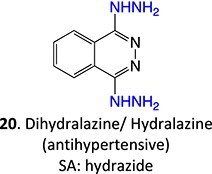	Diazonium	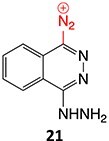	Required	CYP1A2 and CYP3A4	Autoimmune hepatitis	^73^ ^,^ ^82^ ^,^ ^83^
9.	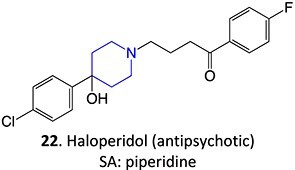	Pyridinium	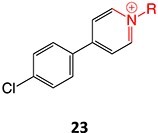	Required	CYP3A4	Parkinsonism, tardive dyskinesia	^84^ ^,^ ^85^
10.	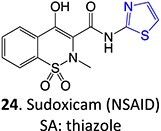	Acyl thiourea Thioamide	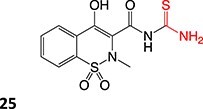 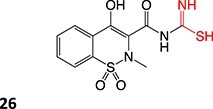	Required	CYP2C8	Hepatotoxic (oxidize protein and glutathione)	^86^ ^,^ ^87^
11.	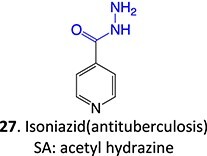	Acetyl hydrazine	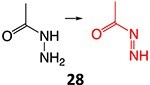	Required	NAT, Amidase, CYP2E1	Hepatitis	^88^ ^,^ ^89^
12.	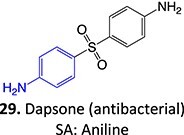	Hydroxylamine	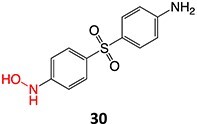	Required	CYP2C8/9	Methemoglobinemia, agranulocytosis, aplastic anemia, cutaneous ADRs	^90–92^
	*Other drugs that form a hydroxylamine:* sulfamethoxazole,^93^ metronidazole,^94^ dantrolene,^95^ flutamide,^96^ nomifensine^62^						
13.	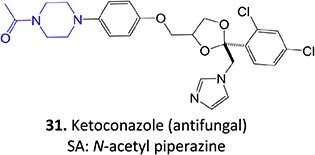 *Another drug that forms deacetylated:* rifampicin^97^	*N*-deacetylation Protonated amide	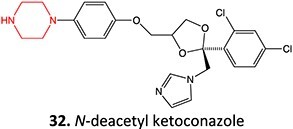 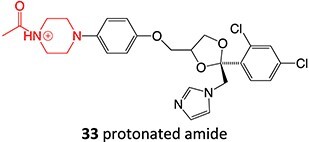	Required	CYP3A4	Undergoes further metabolism by flavin-containing monooxygenase (FMO) to form toxic dialdehyde	^98^ ^,^ ^99^
14.	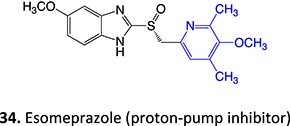	a. Sulfenic acid b. Sulfenamide	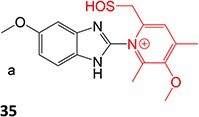 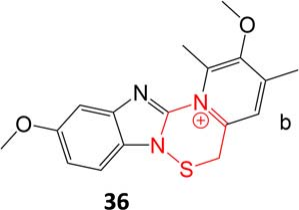	Required	Gastric ATPase	Reacting irreversibly with an active site cysteine in gastric ATPase to form an adduct and leads to inactivation of the proton pump	^100^
15.	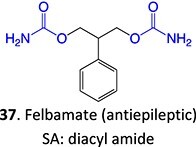	2-Phenylpropenal α, β-Unsaturated aldehyde	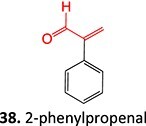	Required	CYP2E1, CYP3A4, CYP2C19	Hepatotoxic, protein alkylation (macromolecule binding)	^101^ ^,^ ^102^
	Other drugs that form an α, β-unsaturated aldehyde: tienilic acid,^100^ trovafloxacin^103^						
16.	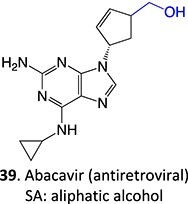	Unconjugated aldehyde	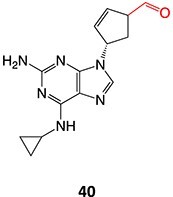	Required	CYPs	Covalent binding to liver cytosol, hypersensitivity	^104^
	*Other drugs that form unconjugated aldehyde:* tranylcypromine,^105^ terbinafine,^106^ zimelidine^107^						
17.	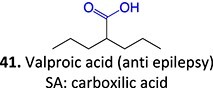	Michael acceptor	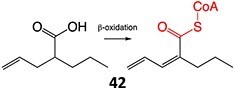	Required	CYPs	Hepatotoxicity (leading to fatalities, considerable risk in children), teratogenicity	^108^
	Another drug that forms a Michael acceptor: terfenadine^109^						
18.	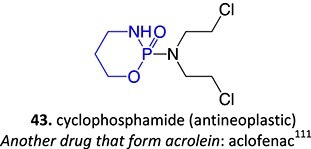	a. Phosphoramide sulfate b. Acrolein	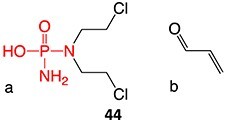	Required	CYP2B6, 2C9, and 3A4	Thrombocytopenia, teratogenic, epidermal necrosis, neutropenia, renal tubular necrosis	^73^ ^,^ ^110^
	*Another drug that forms acrolein*: aclofenac^111^						
19.	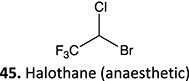	Acyl halide	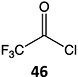	Required	CYP2E1	Hepatitis (immune-mediated)	^73^ ^,^ ^84^
	*Other drugs that form an acyl halide:* isoflurane, desflurane						
20.	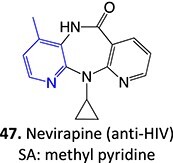	a. Sulfonate b. Imino quinone methide	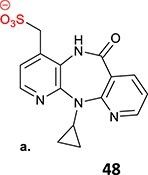 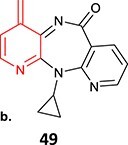	Required	CYPs	Hepatotoxic, skin rash	^67^
21.	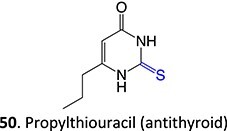	Sulfenylchloride/sulfonate	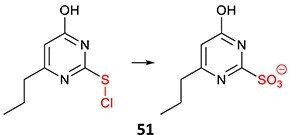	Required	CYPs	Immunological reaction	^68^
22.	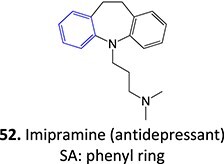	Phenol (NAPQI precursor)	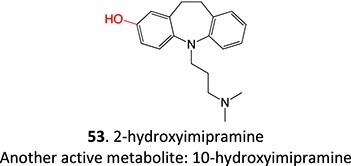	Required	CYP2D6	Hepatitis, agranulocytosis	^112^
	*Other drugs that form a phenol*: clomipramine, venllafaxine^112^						
23.	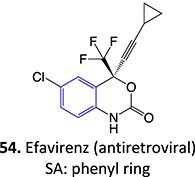	Phenol (NAPQI precursor)	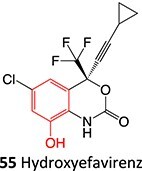	Required	CYP2B6	Inhibiting bupropion hydroxylation in human liver microsomes	^113^
	*Other drugs that form a hydroxyl:* ibuprofen (simvastatin, atorvastatin, lovastatin)^114^						
24.	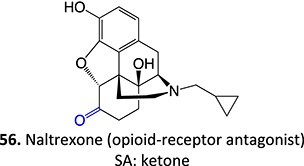	Reduction to alcohol	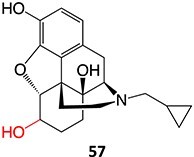	NA	CYPs	Hepatotoxicity	^100^
25.	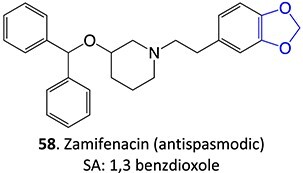	Catechol	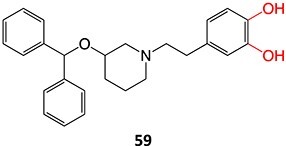	NA	CYPs	Transaminase elevation	^59^
	Other drugs that form a catechol: tadalafil,^37^ carvedilol,^36^ paroxetine^33^						
26.	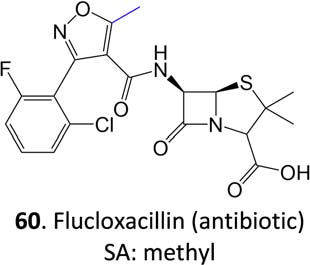	Hydroxyl methyl	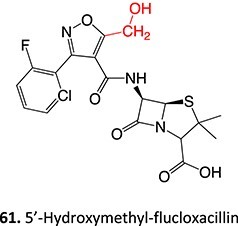	Required	CYP3A4	Cytotoxicity to biliary epithelial cells	^115^
27.	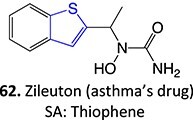	*S*-oxide	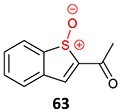	May occur	CYP2E1/ CYP1A2	Transaminase elevation	^116^
	*Other drugs that form an S- oxide:* methimazole,^117^ metiamide,^118^ penicillamine,^119^ tienilic acid,^120^ ticlopidine,^121^ tolrestat,^122^ vicagrel^26^						
28.	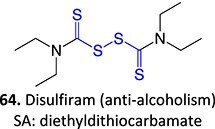	*N, N-*diethylthio-carbamoyl sulfoxide	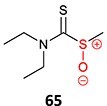	Not required	CYP2E1	Inactivates human protein 2E1	^123^ ^,^ ^124^ ^,^ ^125^
30.	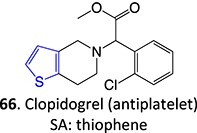	Sulfenic acid	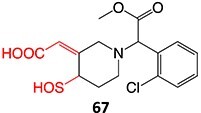	Required	CYP2C19, CYP3A4	Inhibiting the P2Y12 receptor on platelets	^126^ ^,^ ^26^
31.	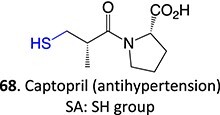	Disulfide	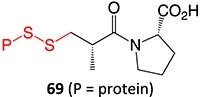	NA	NA	Epidermal necrosis, neutropenia, agranulocytosis	^127^
32.	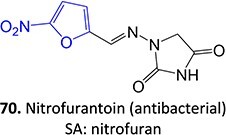	Nitrofuran radical anion to form nitroso	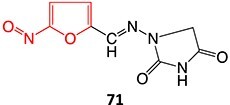	Required	CYP2A6 and CYP3A4	Damage to hepatocytes	^128–130^
33.	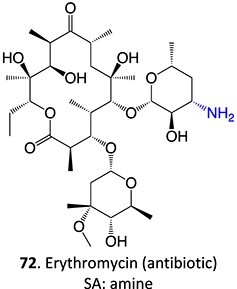	Nitroso	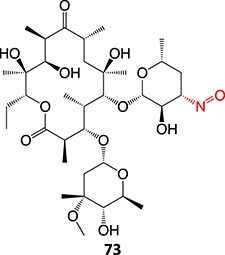	Required	CYP3A4	Forming a complex with the iron ion of CYP3A4’s heme and quasi-irreversibly inhibits the enzyme in a mechanism-based inactivation manner	^131^ ^,^ ^132^
	*Other drugs that form Nitroso:* abiraterone^132^*(*procainamide, sulfamethoxazole),^68^ dihydralazine^8^						
34.	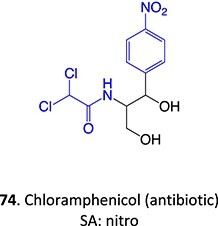	a. Nitroso b. Acyl halide	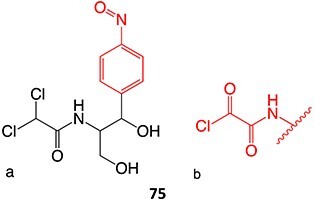	Required	CYPs	Aplastic anemia, cutaneous ADRs/systemic use prohibited	^133^
Epoxides derived from CYP-mediated oxidation of aryl rings, double/triple-bond containing compounds							
35.	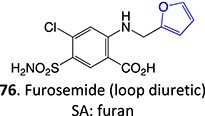	Epoxide	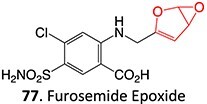	May occur	CYP2C11/ CYP3As /CYP2E1	Midzonal/centrilobular necrosis/focal/multifocal	^134^
	*Other drugs that form an epoxide*: cyclobenzaprine,^135^ (nortriptyline, amitriptyline),^100^ pirprofen,^136^ amineptine,^137^ alpidem,^138^ trazodone,^73^ carbamazepine,^26^ methimazole,^139^ 4-ipomeanol^109^						
36^.^	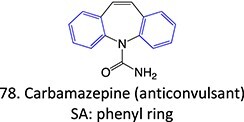	Arene oxide	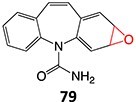	Required	CYP3A4 and CYP2C8	Hepatitis, rash, and agranulocytosis	^73^ ^,^ ^140^
	Other drugs that form an arene oxide: imipramine,^141^ lamotrigine,^142^ phenytoin,^143^ thalidomide^144^						
	Acyl glucuronides						
37^.^	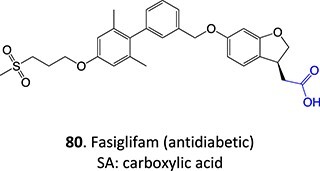	Acyl glucuronide	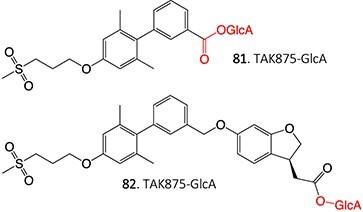	Required	CYPs	Inhibitors of several hepatic transporters	^145^
38.	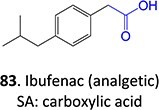 *Other drugs that form an acyl glucuronide:* bromfenac,^146^ benoxaprofen,^147^ zomepirac,^148^ indomethacin,^73^ fasiglifam^149^	Acyl glucuronide	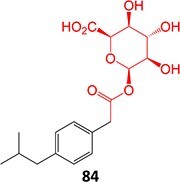	Required	UGT	Hepatitis	^111^ ^,^ ^150^
39	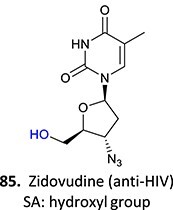	Glucuronide conjugate	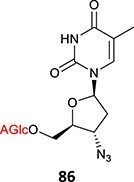	NA	CYP2B, CYP3A, and CYP4A	Toxic to hematopoietic cells	^151^
40	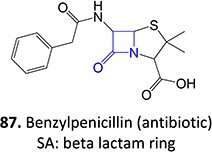	Benzylpenicilloyl	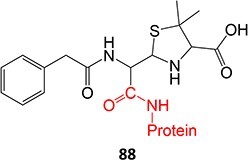	Required	NA	Hypersensitive	^152^

^a^Blue: structural alert; Red: reactive site; NA: not available.

**Fig. 3:**
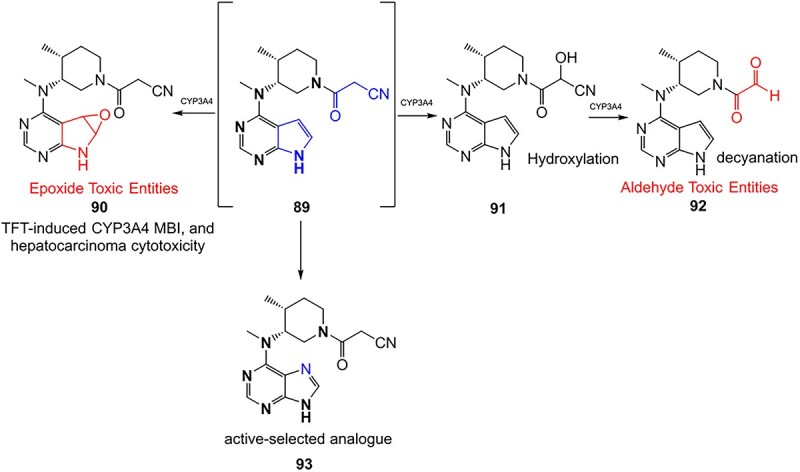
Examples of mitigation of heteroaromatic ring epoxidation via SA replacement. Tofacitinib (**89**) bioactivation to a reactive metabolite.

Strategies that simultaneously mitigate reactive metabolite formation and discover new therapeutic compounds are exemplified by Tateishi and colleagues (Fig. 3).^153^ Tofacitinib (**89**), a non-selective Janus kinase (JAK) inhibitor containing structural alerts (SA), forms toxic metabolites via bioactivation in the liver. The intermediate products **90** and **92** are involved in severe liver injury and associated with a black box warning (BBW) for idiosyncratic adverse drug reactions. Mitigation of heteroaromatic ring epoxidation at the pyrrole double bond in** 89 **was achieved by changing the CH to a nitrogen in **93**. The JAK3 inhibitory activities of compound **93** were weaker, with IC_50_ values ~10-fold higher than compound **89**, 40, and 3.8 nM, respectively. Nevertheless, no evidence of CYP3A inhibition or toxicity toward TC-HepG2 was found in its safety profile, and no adduct formation with *Cys-Glu*-*Dan*, a fluorescent-labeled trapping reagent, was detected. The redesign of 89 successfully mitigated metabolic activation by a structural modification to form the purine analog **93**. Even though the IC_50_ value of **93 **was not equipotent to **89**, the activity was sufficient. The absence of reactive metabolite liability led to safety improvements and a promising candidate to be developed as a JAK inhibitor.

Wurm and colleagues investigated a strategy to mitigate the formation of toxic quinone diimine species.^154^ The adverse effects of the potassium channel openers (K_V_7), flupirtine (**94**), and retigabine (**95**) led to their withdrawal from the market due to the formation of the azaquinone diimines or quinone diimine toxophores (**96**). The reactive metabolites generated from **94** and **95** undergo covalent binding with endogenous macromolecules (**97**), resulting in drug-induced liver injury (DILI). In association with melanin, **96** undergoes dimerization to afford a phenazinium structure (98), causing blue tissue discoloration. The modified lead structures (**99** and **100**) involved displacing the nitrogen atom involved in forming both *ortho*- and *para*-quinone diimines. Among the synthesized analogs tested for activity against HEK293 cells overexpressing the K_V_7.2/3 channel, **101** demonstrated potent K_V_7.2/3 opening activity with an EC_50_ = 310 nM, 6-fold lower than that of flupirtine. The additional methyl group of **101 **may play an important role in this activity. However, its poor water solubility hampered further development.

**Fig. 4:**
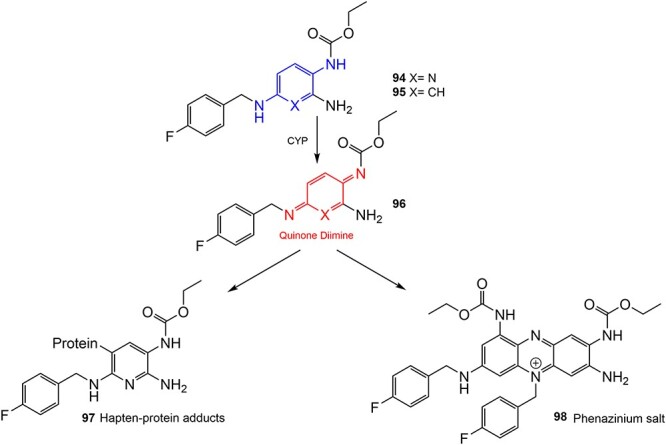
Flupirtine (**94**), retigabine (**95**), and bioactivation to reactive metabolites.^156^

**Fig. 5:**
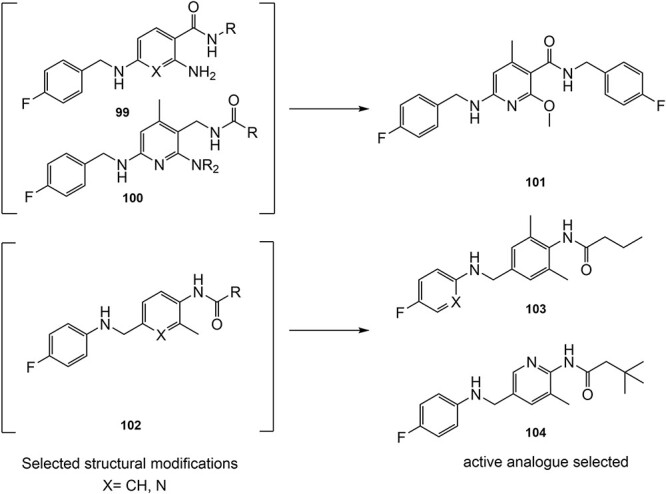
Examples of mitigation of the formation of electrophilic quinone–diimine via SA replacement.

Another study by Wurm and co-workers to attenuate the toxicological properties of **94** and **95** as a potential treatment for pain and epilepsy (Fig. 4).^154^ The goal of this study was to mitigate the quinone diimine or azaquinone diimine metabolite formation. A key approach was triaminoaryl replacement, which is particularly vulnerable to oxidation (**102**, Fig. 5) with alkyl substituents. The analogs (**103** and **104**) demonstrated sub-micromolar activity with up to a 13-fold increase in potency and up to 176% increase in efficacy, compared with **94**. Moreover, the absence of toxicity in vitro indicated that the designed analogs demonstrated better oxidation stability and were not predicted to form quinone metabolites in silico.

**Fig. 6:**
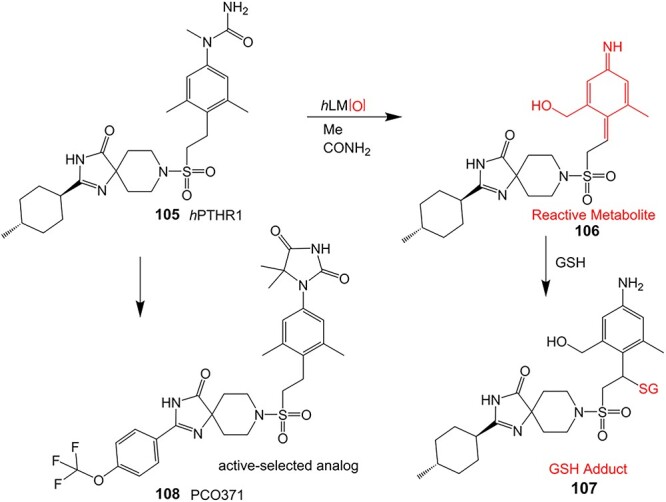
Mitigation of the formation of electrophilic quinone–diimine via SA replacement. *h*PTHR1 (**105**) and bioactivation to reactive metabolites.

A hit-to-lead modification of a novel agonist of parathyroid hormone receptor 1, *h*PTHR1 (**105**), was investigated by Nishimura and colleagues (Fig. 6).^155^ Their findings revealed that this compound tends to form reactive-quinone imine metabolites (**106**), which following hydrolysis and oxidation yield the GSH adduct (**107**) in human liver microsomes. Optimization of the cyclohexyl ring and *N*-methyl urea moiety to prevent undesired metabolites gave **108** as an active-therapeutic analog. During this investigation, **108** showed efficacious *h*PTHR1 agonistic activity, which was metabolically stable, and no GSH adduct formation was detected in human liver microsomes. In addition, the pharmacokinetics and pharmacodynamic profiles also performed as expected; including increased serum calcium and decreased serum phosphate in total parathyroidectomy (TPTX) rats administered orally and dose-dependently with the improved analog.

The above investigations provided metabolically stable analogs that could become viable therapeutic candidates. In addition, no preclinical study of the effect of GSH adduct formation with **105** and no evidence of the toxicity effect of **106** led to research to understand their pathologies. Hence, scaling up in vivo or in vitro metabolite synthesis to milligram levels for toxicology study is required to satisfy the needs of drug development.

ES to produce phase I and II drug metabolites directly could be an option to tractably produce a purified metabolite for downstream toxicology studies.

## ES of drug metabolites

Methods to make drug metabolites directly from the parent drug are limited and often involve multi-step synthesis, typically laborious and time consuming due to the difficulty inherent in synthesizing complex metabolite structures. However, biological methods to generate drug metabolites at a whole cell, subcellular fraction, or animal model enable the estimation of the fate of drugs in the body.^157^ However, these biological methods are not able to provide preparative quantities of drug metabolites directly.

Further limitations of biological metabolite generation methods include (i) binding to cellular macromolecules, (ii) conversion to phase II metabolites, (iii) matrix complexity, (iv) low concentration, (v) limited proliferative potential of isolated hepatocytes, (vi) unstable and short lifespan of primary culture and enzymes, and (vii) limited reproducibility of liver chromosomes,^158–160^ which are also problematic for analyses thereafter.

In addition, reference standards of drug metabolites are indispensable as authentic samples for structural characterization and detection using advanced-analytical chemistry techniques, e.g. liquid chromatography–mass spectrometry (LC/MS) and quantitative nuclear magnetic resonance (NMR).

Thus, a straightforward transformation of the parent drug to the metabolite could be an expeditious approach for investigating drug metabolism vulnerabilities and toxicological studies. With the ability to deliver a direct oxidative or reductive metabolite, ES could come into its own as a drug metabolite generation strategy.^11^ Representative examples of reactive metabolites generated through ES are listed in Table 2. ES can be used in multiple ways including (i) as a metabolite prediction tool by using voltammetric analysis, (ii) as a direct synthetic method to a drug metabolite, and (iii) for the analytical study of oxidative drug metabolism mechanisms when coupled to MS techniques.

**Table 2:** Representative examples of electro-metabolism drug classes.

#	Drugs Ref (s).	Reaction types	ES products	ES conditions	In vivo biotransformation	Ref (s).
1.	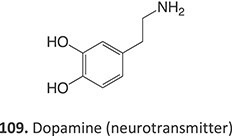	Quinone formation	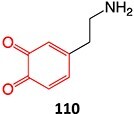	Potential 150 mV divided cell, WE: graphite, c.e.: graphite, RE: S.C.E. pH 7.2 phosphate buffer	Yes	^161^
2.	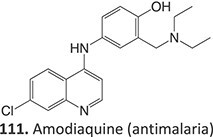	Quinone imine formation Aldehyde formation	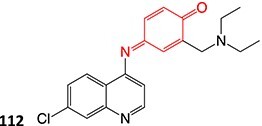 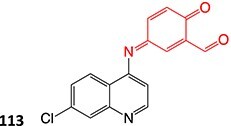	Buffer containing 50/50 (v/v) 100 mM aqueous ammonium formate (pH 7.4)/acetonitrile, WE: Platinum, CE: graphite-doped Teflon, RE: palladium/hydrogen, potential sweep: 0–2000 mV, scan rate: 10 mV/s. or Potential 1000 mV WE: carbon, CE: Pt wire, RE: Ag/AgCl .1 M phosphate buffer	Yes	^162^ ^,^ ^163^
						
3.	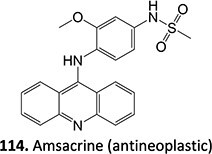	Quinone diimine formation	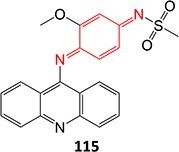	Potential 300 mV flow cell WE: glassy carbon CE: Pd, RE = Pd/H_2_ MeCN NH_4_CO_2_H/NH_4_OH pH 7.4	NA	^164^
4.	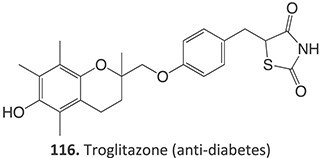 Other drugs that form quinone methide formation via ES: toremifene, ^165^^,^^166^ nevirapine^167^	Quinone methide formation	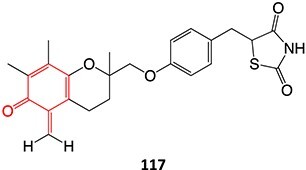	Potential 200 mV, WE: porous graphite, CE: Pd, RE: Pd/H_2_, 0.1 M phosphate buffer (pH 7.4)/acetonitrile (3:1 v/v)	Yes	^168^ ^,^ ^169^
5.	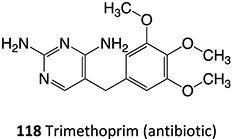	Imine methide formation	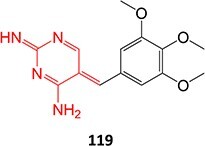	Potential 600 mV, flow cell, WE: carbon, CE: Pt wire RE: Ag/AgCl 0.1 M phosphate buffer	NA	^73^ ^,^ ^165^
6.	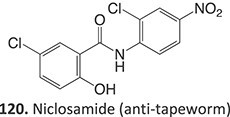	Nitroso formation	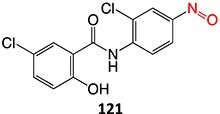	Potential: 0–2500 mV scan rate: 5 mV/s flow cell WE: boron-doped diamond CE: not stated RE: Pd/H_2_, MeOH NH_4_OAc	NA	^170^
7.	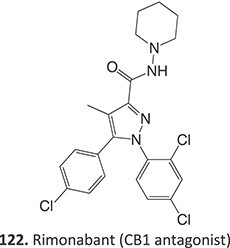	Aldehyde formation	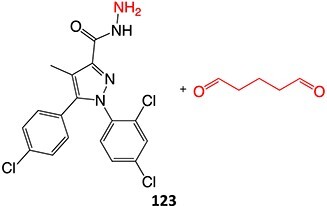	Potential 0–1000 mV, MeCN/HCO_2_H/H_2_O, WE: porous graphite, RE: Pd	Yes	^171^
8.	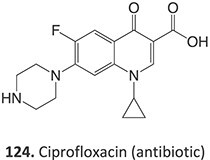 Other drugs that form an iminium ion: chlorpromazine, clozapine,^162^ haloperidol^11^^,^^172^	1-hydroxypiperazin-1-ium formation	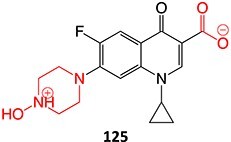	WE: glassy carbon electrode (GCE), auxiliary electrode platinum rod, RE: saturated calomel electrode (SCE), 0.04 M Britton– Robinson (BR) buffer solution pH 7, scan rates from 10 mV/s to 500 mV/s, Potential ranges −200 to +1500 mV	NA	^173^
9.	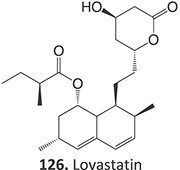 Other drugs that undergo hydroxylation: simvastatin,^174^ tetrazepam,^175^ triclocarban,^176^ tolterodine,^177^ testosterone,^178^ cocaine^179^	Hydroxylation	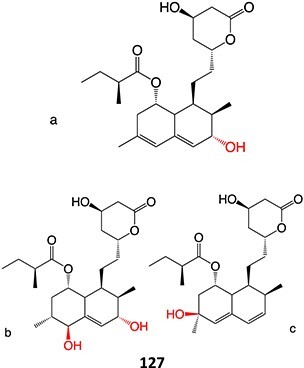	Ammonium acetate buffer in water– acetonitrile (1:1 v/v) at a pH of 7.0, 1500 mV, WE: glassy carbon (GC) or a boron-doped diamond (BDD), RE: Pd/H_2_	Yes	^174^
10	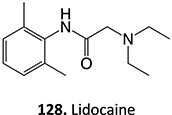	a. Aromatic hydroxylation b. Dealkylation c. Arene-oxide formation	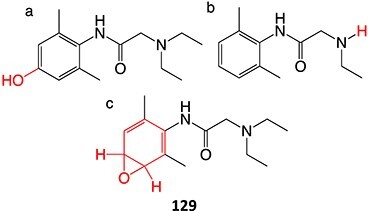	a, b. SW-voltammetry, Platinum electrode 5000 mV, TBAP, ACN/H_2_O c. Platinum electrode, 3000 V, acetonitrile/ water 99/1 (v/v), .1 M TBAP	Yes	^180^ ^,^ ^181^
11.	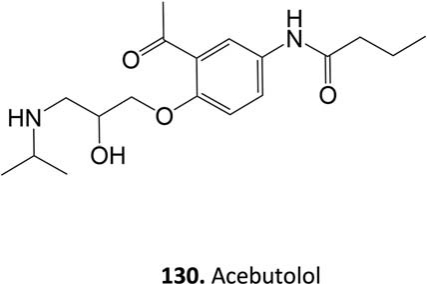 Other drugs that undergo dealkylation: alprenolol,^182^ mephenyotin, metoprolol,^178^ toremifene,^166^ boscalide,^183^ berberine^184^	a. *N*-dealkylation b. Benzyl hydroxylation c. *O*-dealkoxylation	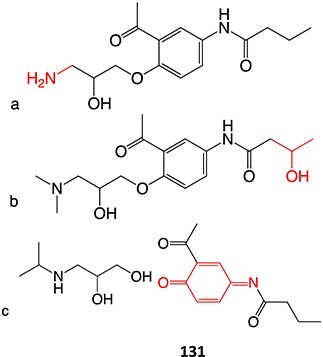	NH_4_OAc (aq) / acetonitrile (1/1), flow rate of 0.5 mL/min, potential 1000 mV, WE: Carbon, RE: Pd/H_2_	Yes	^182^
12.	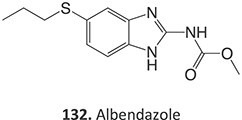 Another drug that form an iminium ion: chlorpromazine^185^	*S*-oxidation	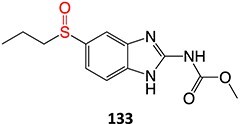	I:24 mA, 1.5 *F*/mol, 3:1 MeCN/H_2_O HCl	NA	^185^
13.	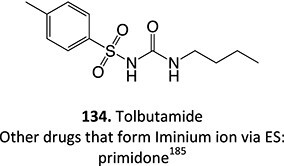 Other drugs that form an iminium ion: primidone^185^	Methylene-oxidation	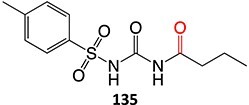	1300 mV, RE: SCE, 6 *F*/mol, 1:1 MeCN/H_2_O NaHCO_3_	Yes	^185^
14.	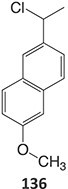	Carboxylation	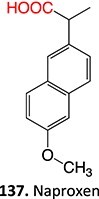	Undivided cell, CO_2_, solvent: tetramethyl urea (TMU), Potential range −1800 to +2500 mV, WE: glassy carbon, CE: platinum, RE: saturated calomel electrode (SCE)	NA	^186^
15.	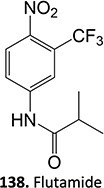	Hydroxylamine	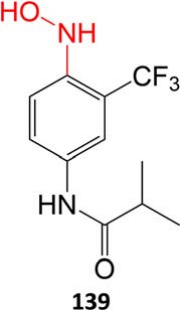	WE: boron doped diamond, Ref electrode: Ag/AgCl, 40 mM Britton-Robinson pH 2.25 0.1 M H_2_SO_4_, potential range from −50 to +2200 mV with the scan rate of 100 mV/s	Yes	^187^ ^,^ ^188^
16.	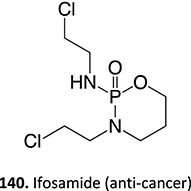 Another drug that forms Iminium ⍺-methoxy metabolite : cyclophosphamide^189^	⍺- methoxy metabolites	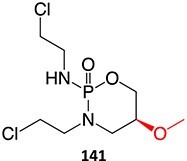	Glassy carbon electrode, 1850 and 2000 mV, methanol/ Et_4_NOTs, *I*: 15 mA, 2.2 *F*/mol	Yes	^189^
17.	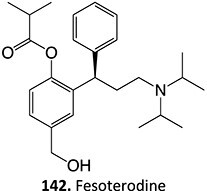	a. Dealkylated metabolites b. Deamination	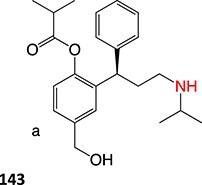 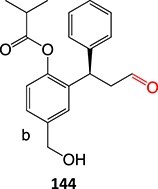	WE: tubular reticulated glassy carbon, RGC, RE: Pd/H_2_ HyREF, auxiliary electrode: coiled platinum wire, scan rate: 20 mV/s, ammonium acetate solution, potential 950 mV.	NA	^190^
18.	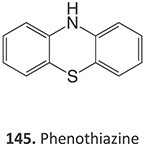	Dimer formation	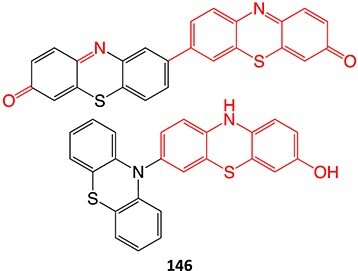	Undivided cell, a mixture of water (phosphate buffer, pH 3.0, *c* = 0.2 M) acetonitrile (50/50 v/v), WE: glassy carbon electrode, CE: platinum, at scan rate of 10 mV/s, room temperature	No	^191^
19.	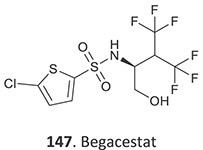	*N*-dealkylation	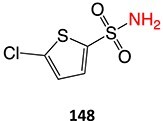	LiClO_4_, MeCN–MeOH (9:1), *l*: 20 mA, *j*: 0.50 mA/cm^2^, *Q* = 4.0 *F*/mol, RVC(+) RVC(−)	NA	^192^

NA, not available; WE = working electrode; CE = counter electrode; and RE = reference electrode.

**Fig. 7:**
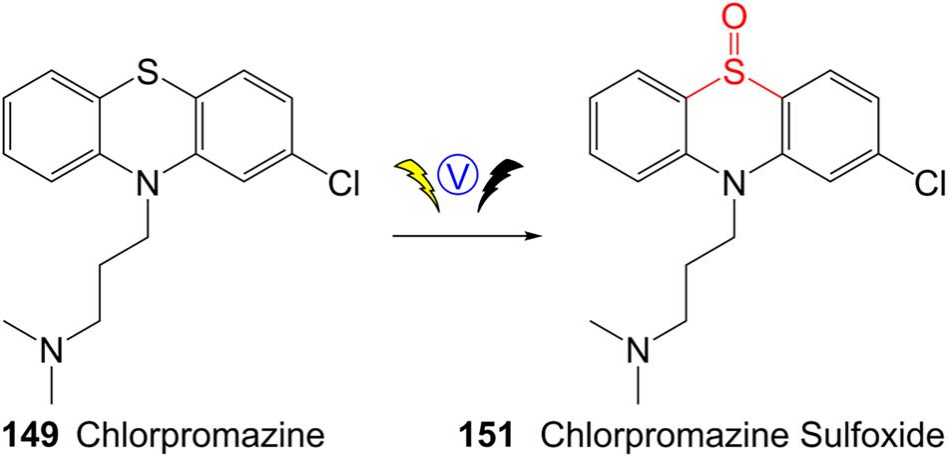
Traditional synthesis of chlorpromazine metabolites. Electrochemical conditions: GNs–CdS QDs/IL/CPZ modified GC electrode in 0.1 M PBS (pH 7.0) at a scan rate of 100 mV/s, potential range −200 to +400 V, RE: Ag/AgCl/KCl (3.0 M), CE: platinum wire, WE: GC (modified and unmodified). GNs–CdS QDs/IL/CPZ modified GC electrode: a nanocomposite containing graphene nanosheets and CdS quantum dots (GNs–CdS QDs).

**Fig. 8:**
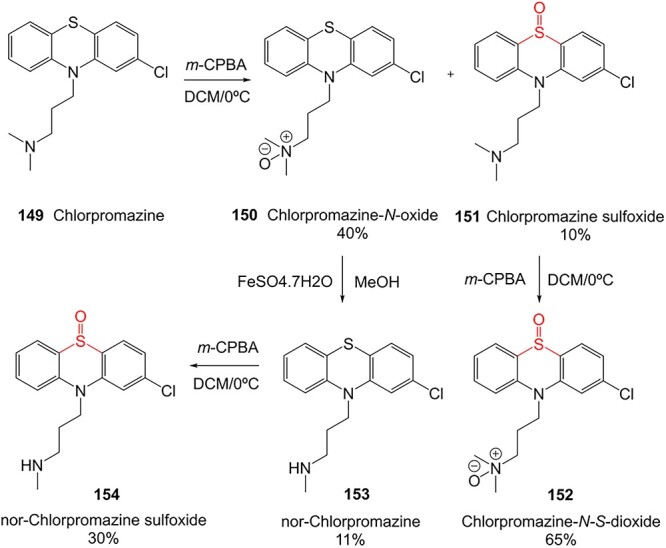
ES of chlorpromazine metabolites.

The direct ES of a drug can be an efficient alternative for the synthesis of a complex metabolite compared with a multi-step chemical synthesis approach. Jafari and co-workers developed a simple electrochemical oxidation of chlorpromazine to chlorpromazine-sulfoxide (Fig. 7).^193^ In contrast to what Kigondu and colleagues^194^ found in synthesizing the same metabolite, a non-ES method required a multi-step process to afford the metabolite via a non-classical Polonovski reaction (Fig. 8). Even though a small number of corresponding metabolites were detected in step 1, it still required further steps to scale up the product.

**Fig. 9:**
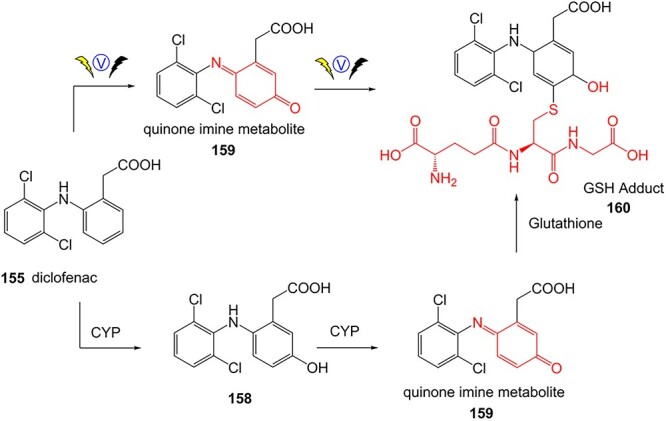
The comparison between biological study^195^ vs. ES technique of diclofenac.^196^*ES conditions:* Diclofenac (50 μM) in 5.0 mM NH_4_OAc (pH 7.4)/ACN 50/50 v/v, WE: boron-doped diamond, potential 0–2500 mV within 250 s. GSH adduct formation at potential = 2400 V, GSH 50 °C 1:1 acetonitrile/water.

**Fig. 10:**
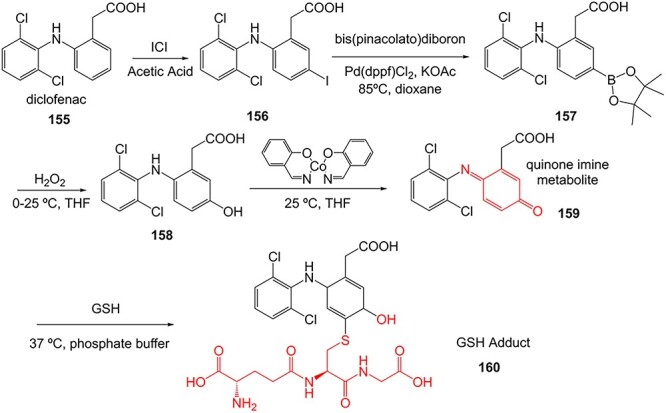
Traditional synthesis of a diclofenac–GSH adduct.^195^

A further example of the use of electrochemistry (EC) revealed the simplicity of transforming diclofenac to a quinone imine metabolite (Fig. 9).^185^^,^^196–198^ Diclofenac, a nonsteroidal anti-inflammatory drug (NSAID), was reported to have DILI associated with the formation of reactive metabolites at higher accumulation. In humans, CYP2C9 and CYP3A4 bioactivate diclofenac to yield 4-hydroxydiclofenac and 5-hydroxydiclofenac and undergo further oxidation to form reactive quinone–imine intermediates, trapped by GSH resulting in glutathione adducts (Fig. 10). The inherent advantage of ES enabled a simple and fast preparation of metabolites directly from the drug molecule in comparison to traditional bespoke syntheses or biological studies.

## Applications of ES to toxicology studies

**Fig. 11:**
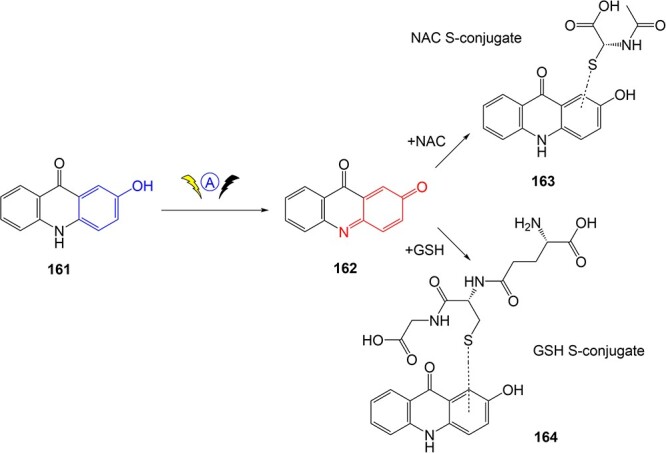
Metabolism simulation of antitumor-active 2-hydroxyacridinone. (161, 2-OH-AC). *ES conditions:* electrochemical thin-layer cell; WE: disc glassy carbon (GC); RE: Pd/H_2_; flow rate of electrolyte 30 μL/min; potential ranges 0–2500 mV; scan rate 10 mV/s; electrolyte 0.1% formic acid in water/methanol (50:50 v/v).

Potęga and co-workers^157^ demonstrated metabolism mimicry of 2-hydroxy-acridinone (2-OH-AC), **161**, a reference compound for antitumor-active triazoloacridinone derivatives (Fig. 11). Using an electrochemical thin-layer cell system in tandem with MS, **161** was converted to the reactive quinone imine oxidation product (**162**) and trapped via conjugation with nucleophilic agents such as glutathione and *N*-acetylcysteine (NAC) as biomarkers of metabolic activity in phase II metabolism. This electrochemical process generated metabolite adducts, NAC *S*-conjugate (**163**) and GSH *S*-conjugate (**164**), through the covalent bond with the thiol group. **164** was also found in the human and rat liver microsomes through enzymatic experiments. This study generated numerous different products and enabled structural diversification and modification. Further research is required to determine whether this quinone–imine metabolite contributes to the toxicity of **161** in vivo, as the metabolite–adduct formation is not necessarily indicative of toxicity.^199^

**Fig. 12:**
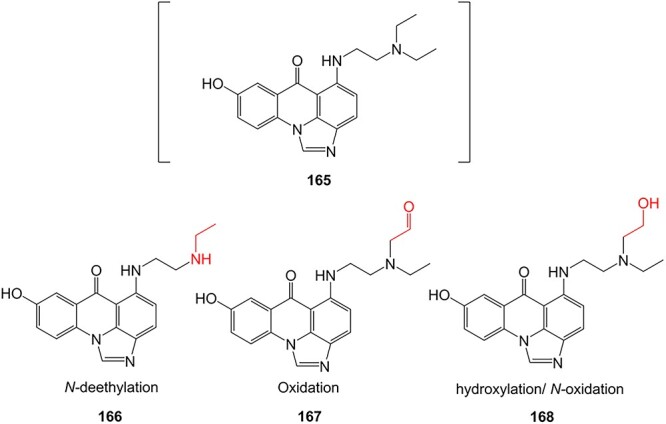
Phase I metabolism simulation of 5-diethylaminoethylamino-8-hydroxyimidazoacridinone. *ES conditions:* H_2_O–MeOH (1:1, v/v) pH 3.3 and NH_4_ HCO_2_–ACN (1:1, v/v) pH 7.4, WE: GC, RE: HyREF palladium-hydrogen (Pd/H_2_), an auxiliary electrode: carbon-loaded polytetrafluoroethylene, flow rate 30 μL/min, potential 0–2500 mV (10 mV/s).

5-Diethylaminoethylamino-8-hydroxyimidazoacridinone (165, C-1311), a novel antimetastatic compound for breast cancer, was electro-metabolized by Potęga and colleagues (Fig. 12). Derivatives were generated via *N*-dealkylation, dehydrogenation, hydroxylation, and oxidation reactions.^200^ Coupling EC with electrospray ionization–MS (ESI–MS), the authors simulated phase I metabolism of **165** and demonstrated agreement with the metabolite generation in the in vivo*/*in vitro models and in silico prediction of the metabolism site (**166**–**168**). The electrochemical method revealed other metabolites not seen in other metabolic studies and enabled structural diversification.

**Fig. 13:**
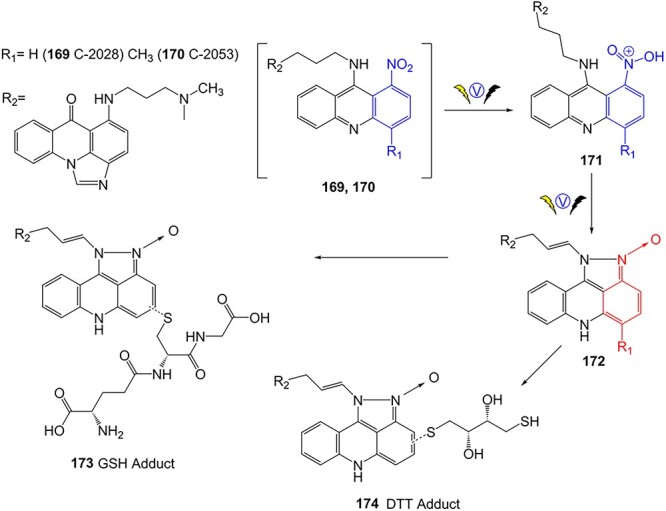
The reactive heterocyclic di-*N*-oxide metabolite and adduct formation of C-2028 and C-2053 via electro-metabolism simulation. *ES conditions:* electrochemical thin-layer reactor cell; WE: disc GC or boron-doped diamond (BDD); RE: HyREFTM palladium-hydrogen (Pd/H_2_); auxiliary electrode: carbon-loaded polytetrafluoroethylene; electrolyte H_2_O–MeOH (1:1, v/v) with 0.1% FA; flow rate of electrolyte 20 μL/min; potential ranges −1500 to −500 mV and −2500 to −1500 mV; scan rate 5 mV/s; and *T* = 21 °C.

Potęga and colleagues replicated the phase I and II metabolism products of novel disparate antitumor classes on a preparative scale, with the unsymmetrical bisacridine antitumor agents C-2028 (**169**) and C-2053 (**170**) (Fig. 13).^201^ These compounds underwent an EC process coupled with LC–MS, enabling the detection of their metabolites, respectively. In this study, the SA of the nitroaromatic moiety is susceptible to reductive transformation affording the stable hydroxylamine, amine, and *N*-oxide products. However, the heterocyclic di-*N*-oxide metabolite (**172**) could become reactive under oxygen-depleted conditions, which might be responsible for the antitumor activities or degradation of cellular biomolecules. In the phase II metabolism step, the C-2028 metabolite was trapped via GSH and DTT, which generated the metabolite-adducts **173** and **174**.

Compared to **169**, a metabolite adduct of **170** was not detected in this study. The *para* position to the nitro group is hypothesized to be the most likely conjugation site with GSH or DTT. Thus, the existence of the R_1_ = methyl group in **170** could diminish its susceptibility to interactions with trapping agents.

**Fig. 14:**
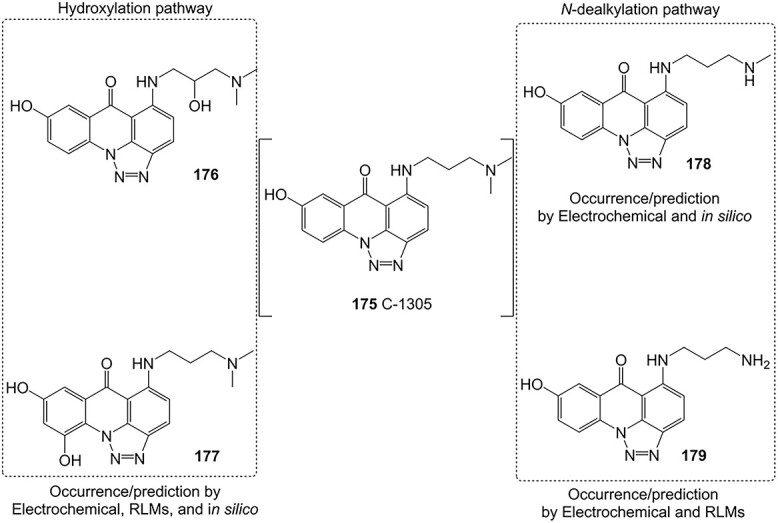
Phase I metabolism simulation of 5-dimethylaminopropylamino-8-hydroxytriazoloacridinone (C-1305). *ES conditions:* WE: GC, flow rate 20 μL/min, potential 0–2500 mV (10 mV steps), electrolyte: 0.1% HCO_2_H in water/CH_3_OH (50:50, v/v).

To predict oxidative pathways, Potęga and co-workers also revealed the metabolic transformation of 5-dimethylaminopropylamino-8-hydroxytriazoloacridinone (**175**, C-1305), a triazoloacridinone antitumor derivative (Fig. 14).^202^ Multi-tool approaches, e.g. electrochemical setup, rat liver microsomal model, and in silico analysis, were used to predict the generated metabolic products of C-1305 in phase I metabolism. In this study, the dialkylaminoalkylamino moiety of **175** was found to be susceptible to oxidative transformation via *N*-dealkylation, dehydrogenation, and hydroxylation, which may be responsible for cytotoxic and antitumor actions of C-1305 metabolites. ES revealed similarities in relation to several metabolites generated via incubation with rat liver microsomes (**176**–**179**). These findings demonstrated that ES can be used to expedite the drug development process.

**Fig. 15:**
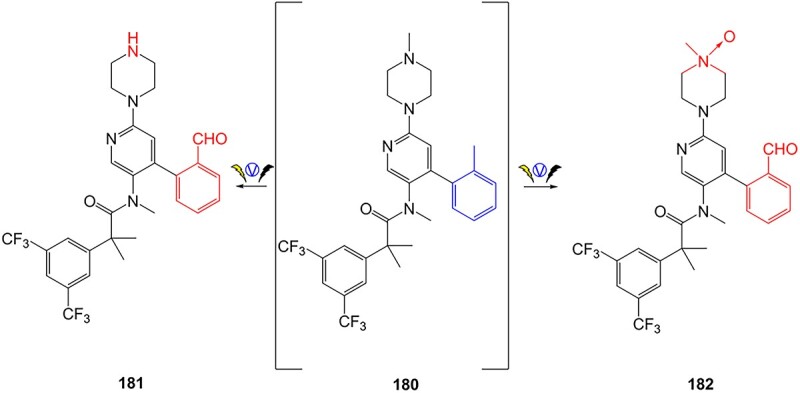
Phase I metabolism simulation of netupitant. *ES conditions:* flow rate: 15 mL/min, WE: boron-doped diamond, CE: conductive polyether ether ketone, RE: Pd/H_2_, potential: 0–2500 mV, scan rate of 10 mV/s.

Chira and co-workers have reported a metabolism product of netupitant (**180**, an NK1 receptor antagonist) via a controlled potential EC coupled with MS (Fig. 15).^203^**180** was electro-oxidized, resulting in a significant number of hydroxylated, dehydrogenated, alkylated, and *N*-dealkylated metabolites that occurred both in vivo and in the electrochemical biotransformation. Among the metabolites generated, a benzaldehyde was generated in **181** and **182** via oxidation to a carbonyl. However, no mechanism of action or the metabolites' fate was reported. The corresponding electrochemically unconjugated aldehyde-containing metabolite can be speculated to initiate some detrimental effects, which may form covalent bonds to nucleophilic sites of DNA, leading to carcinogenicity.^204^ This study did not find evidence of the mono *N*-demethylated product as a major metabolite of netupitant.

Netupitant is an antiemetic medication that has been approved by the FDA, in combination with palonosetron, to delay chemotherapy-induced nausea and vomiting.^205^ Due to the dearth of information about the metabolites’ structures, and the possible toxic generation of these drug metabolites that may occur during biotransformation is a cause for concern. Therefore, additional structural elucidation to assist a comprehensive safety study, such as in vivo or in vitro studies, may be needed to generate a novel derivative.

**Fig. 16:**
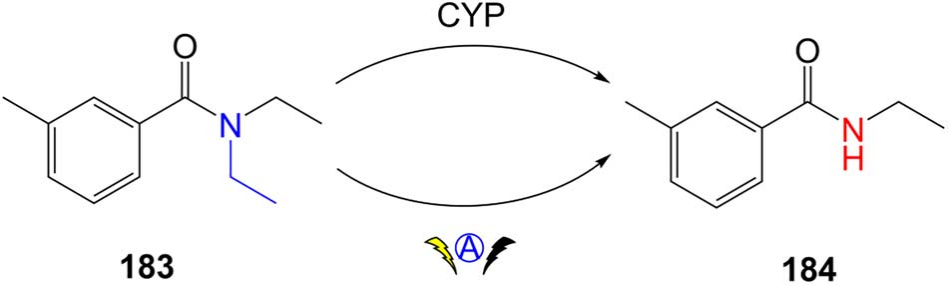
Metabolism mimicry of DEET. *ES condition*: controlled current conditions, reticulated vitreous carbon (+RVC / –RVC), 0.5 M Bu_4_NClO_4_, MeCN:MeOH (10:1) 0 °C, *Q*: 4 F/mol, *l*: 5.0 mA, current density: 0.71 mA/cm^−2^.

Metabolism mimicry using ES was employed by Bal and colleagues (Fig. 16).^206^ Conversion of diethyltoluamide (DEET, **183**), a common active ingredient in insect repellents, enabled the preparation of **184** the primary human metabolite of DEET. This study highlights the potential of ES as a method for preparing human metabolites on a preparative scale.

## Conclusions

The similarities between ES-generated and enzymatically generated metabolites have provided new insight into the origins of drug bioactivation pathways by mimicking phase I and II metabolisms. In this review, we have showcased the applications of ES for drug metabolism studies, including the ability to identify reactive or toxic metabolites for an NCE; the use of this information to mitigate metabolism via SA alteration; and the use of ES to enable rapid late-stage diversification of drug candidates. Key advantages of ES are that preparative samples of the desired drug metabolite are directly obtained from the parent drug; ES is often much simpler compared to traditional routes; and green ES uses mild conditions with limited use of additional chemicals/solvents.

Although not the focus of the review, ES can be combined with LC/MS and quantitative NMR for structural characterization and detection to study oxidative drug metabolism in situ. Thus, the usefulness of ES as a complementary approach could play a broader role in future toxicological studies.

## Data Availability

All data associated with the article are contained within the research papers. ***Conflict of interest statement:*** None declared.
